# Genomic Characterization of Three Melon Necrotic Spot Viruses Detected in Human Stool Specimens

**DOI:** 10.1128/genomeA.01758-16

**Published:** 2017-03-16

**Authors:** Rachel Marine, Christina Castro, Laura Magaña, Terry Fei Fan Ng, Kshama Aswath, Nikail Collins, Geun Woo Park, Jan Vinjé, M. Steven Oberste

**Affiliations:** a Division of Viral Diseases, Centers for Disease Control and Prevention, Atlanta, Georgia, USA; b Oak Ridge Institute for Science and Education, Oak Ridge, Tennessee, USA; c CDC Foundation, Atlanta, Georgia, USA; d Atlanta Research and Education Foundation, Atlanta, Georgia, USA

## Abstract

The complete coding sequences of three melon necrotic spot viruses (MNSVs) were obtained from viral metagenomics of stool samples from patients with acute gastroenteritis. These genomes were most similar to Spanish strains sequenced in 2003 and a novel MNSV watermelon strain in 2014.

## GENOME ANNOUNCEMENT

The human gastrointestinal (GI) tract is inhabited by a wide variety of viruses, including enteric viruses, bacteriophages, and plant and insect viruses acquired through diet. Metagenomic analyses of human enteric RNA viruses have shown that plant viruses are highly abundant in gut viromes and remain infectious to plants even after transit through the gut (1). While there is some speculation that plant viruses may interact with human cells or the immune system (2), it is more likely that plant viruses detected in stool are merely a snapshot of infections in commercial crops. Understanding the diversity of plant viruses which pass through the human digestive tract is necessary to gain a complete picture of the gut microbiome and may serve as an indicator for circulating plant pathogens.

Next-generation sequencing (NGS) was utilized to investigate clinical stool specimens from three separate norovirus outbreaks in 2012 (Oregon, USA), 2014 (Maryland, USA), and 2016 (cruise ship outbreak, USA). Because a metagenomic sequencing approach was employed (3), which allowed simultaneous sequencing of multiple pathogens, near-complete melon necrotic spot virus (MNSV) genomes were also coincidentally assembled from three of the samples analyzed. MNSV is a member of the genus *Gammacarmovirus* (family *Tombusviridae*) that infects melons and cucumbers. It is endemic in Japan (4–6), the Americas (7), and Europe (8, 9) and has been reported in Africa (10) and Asia (11). The genome consists of an ~4.3-kb positive-sense RNA encoding five proteins (p29, p89, p7A, p7B, and p42), with short 5′ and 3′ untranslated region (UTR) sequences flanking the coding regions (12). The 3′ region contains the cap-independent translational enhancer element, which is responsible for translation initiation factor recruitment and influences host range (13).

The three sequenced MNSV genomes range from 4,204 to 4,240 nucleotides (nt) and include all coding sequences, with short regions missing at the 5′ and 3′ ends. Genomes assembled from the 2012 and 2014 specimens, MNSV/USA/MD/2012 and MNSV/USA/OR/2014, respectively, are 100% identical to one another despite the stool samples being collected roughly 1.5 years apart and in different states. BLASTn comparison of the MNSV genomes to sequences available in the NCBI database showed that genomes MNSV/USA/MD/2012 and MNSV/USA/OR/2014 shared ≤69.3% nucleotide identity with other MNSV genomes, with the greatest nucleotide identity to watermelon sequence MNSV-Kochi (AB232926). Interestingly, these genomes shared a high degree of identity (95.7%) to a partial genome (2211 nt) of a novel watermelon MNSV strain detected during a 2014 outbreak in a commercial greenhouse in Spain (14). In contrast, the third genome, MNSV/USA/2016, was more similar to previously sequenced MNSV genomes. While the MNSV/USA/2016 genome shares the greatest nucleotide identity (94.9%) to Spanish isolate MNSV-AI (DQ339157), phylogenetic analysis using the p89 gene (RNA-dependent RNA polymerase) indicated a closer evolutionary link to MNSV isolates N (KF060715) and ABCA13-01 (KR094068). Similar to the ABCA13-01 genome (15), the 3′ end of the MNSV/USA/2016 genome does not contain the 55-nucleotide resistance-breaking sequence of MNSV-N (13). Our findings suggested that gammacarmoviruses from plants can pass through the human gut and could be a component of the human gut microbiome.

### Accession number(s).

Genomic sequences for MNSV/USA/MD/2012, MNSV/USA/OR/2014, and MNSV/USA/2016 have been deposited in GenBank under the accession numbers KY124135 to KY124137.
